# An overview of malarial *Anopheles* mosquito survival estimates in relation to methodology

**DOI:** 10.1186/s13071-020-04092-4

**Published:** 2020-05-07

**Authors:** Justin Matthews, Alison Bethel, Goldie Osei

**Affiliations:** 1 College of Medicine and Health, University of Exeter, St. Luke’s Campus, Heavitree Road, Exeter, EX1 2LU UK; 2 NIHR ARC South West Peninsula (PenARC), University of Exeter, Exeter, UK

**Keywords:** *Anopheles*, Mosquito, Malaria, Survival

## Abstract

**Background:**

The transmission of malaria is known to be sensitive to the survival (longevity, mortality) of its mosquito vector, yet there have been few reviews of estimates of this important population parameter in the malaria-carrying genus *Anopheles*.

**Methods:**

We carried out a systematic search for and meta-analysis of survival estimates, framed around the methods of estimation, under the major groupings of ‛vertical’ (based on stable age or stage frequencies), ‛horizontal’ (based on recaptures of marked and released cohorts), and ‛parasitological’ (proportion of infectious mosquitoes). Because of the intricacies of the estimation process we provide an outline of these methods.

**Results:**

By meta-analysis we quantify the average of the distribution of daily survival $p$ for vertical (0.83, 95% CI: 0.80–0.86), horizontal (0.73, 95% CI: 0.66–0.79) and parasitological (0.92, 95% CI: 0.86–0.95) methods.

**Conclusions:**

The meta-analysis demonstrates the anticipated result that horizontal estimates are lowest because they estimate apparent survival (survival and non-emigration) rather than true survival. On the other hand, vertical methods make strong assumptions about the stability or stationarity of the underlying populations. Further potential sources of methodological bias are mentioned. The substantial differences in estimates between methods indicates that methodological biases need to be considered when making use of available survival estimates.
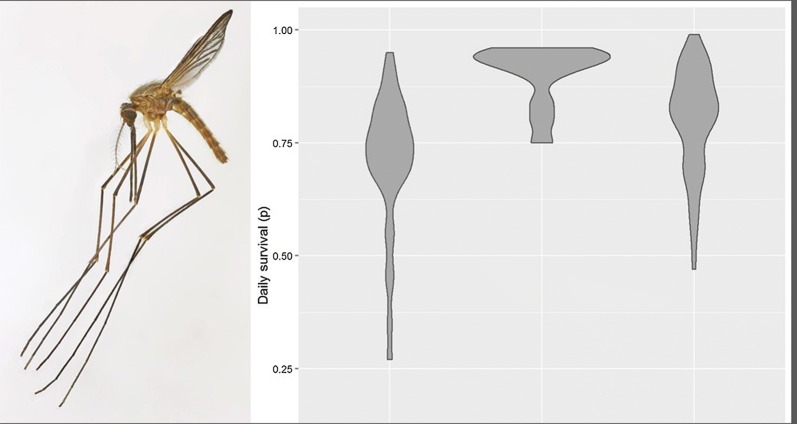

## Background

Only infected mosquitoes that survive beyond the incubation period of the malaria parasite will transmit the disease. Transmission models and intervention effects have been shown to therefore be highly dependent on mosquito mortality [1–3]. But published estimates of mortality or related concepts (longevity, survivorship) of wild mosquitoes are highly variable (species-specific lifespan ranges of 3.6 to 20 days [4]; 5.6–32 days [1]; or median daily survival rates from 0.68 to 0.98 (corresponding to longevity of 2.6 to 50 days [5]). This heterogeneity has not been examined previously in relation to the underlying methodology.

Mosquito lifetime or survival can be measured under laboratory conditions, but here the multifactorial sources of ‛wild’ death including predation, dessication and cold have been eliminated or at least modulated. However, in the wild it is not possible to continuously follow or track individuals (mosquitoes typically weigh less than 10 mg). Neither can samples of wild, dead mosquitoes be feasibly collected in an animal so small, ruling out those ‛mark-recovery’ methods [6] which record the time interval after release when marked animals are recovered dead (used with larger, more visible animals e.g. ringed birds). So other means of inferring survival are required.

Broadly speaking, the methods that have been used to estimate survival or longevity can be divided into three major groups [7]: (i) the rate of disappearance of a marked sample; (ii) inferences from the infectiousness of the mosquitoes; (iii) the age or stage structure of samples.

Horizontal (longitudinal) methods (i) follow a cohort through time while vertical methods (iii) depend on samples taken (conceptually at least) at one moment in time (parasitological estimates (ii) are not so easily characterised).

With reference to their own survival estimates obtained from age frequencies Gillies and Wilkes [8] noted that “Perhaps the most important aspect of these results is the lack of agreement with estimates of survival for *gambiae* and *funestus* derived from less direct methods of analysis such as parous rates, ratio of immediate to delayed sporozoite rates and epidemiological analysis of the sporozoite rate … this discrepancy is so great as to have a marked effect on some aspects of the epidemiology of malaria”. Use of the methods mentioned has continued for half a century, and in this paper we examine whether and by how much this lack of correspondence is still observed.

Our purpose here is to review *Anopheles* survival estimates using a systematic search paying particular attention to the estimation methods used. We bring together the methods that we went on to find, expressed in a matrix discrete-time form that can be applied to either a demographic or disease stage classification.

Previous publications containing collated information on this topic are limited in one way or another: some are narrative [9]; were not systematic (and may not have been designed to be), that is did not lay out their methodology and are not repeatable [1, 4, 10, 11]; provided limited details or results [5, 12]; or were not concerned with *Anopheles* mosquitoes [13]. Some review a subset of the genus (*An. gambiae* [14] and *An. punctulatus* [15]) or were restricted to a particular approach [12].

## Methods

### Vertical methods

#### Matrix population models

We assume a discrete-time model in which the time steps represent a mosquito feeding-oviposition cycle over which survival probability is ($\phi$). In the literature this is related to the probability of daily survival ($p$) by

1 $$p^{d} = \phi$$

where $d$ is the duration of the cycle in days.

***Age-structured matrix*** We first describe the adult mosquito population dynamics in general age-structured matrix form:

2 $$\left[ {\begin{array}{*{20}l} {x_{0} } \hfill \\ {x_{1} } \hfill \\ {x_{2} } \hfill \\ \vdots \hfill \\ {} \hfill \\ \end{array} } \right]\left( {t + 1} \right) = \left[ {\begin{array}{*{20}l} {f_{0} } \hfill & {f_{1} } \hfill & {f_{2} } \hfill & {f_{3} } \hfill & \ldots \hfill & {} \hfill & {} \hfill \\ {\phi_{0} } \hfill & 0 \hfill & 0 \hfill & 0 \hfill & \ldots \hfill & {} \hfill & {} \hfill \\ 0 \hfill & {\phi_{1} } \hfill & 0 \hfill & 0 \hfill & \ldots \hfill & {} \hfill & {} \hfill \\ \vdots \hfill & \vdots \hfill & \vdots \hfill & \vdots \hfill & \ddots \hfill & {} \hfill & {} \hfill \\ {} \hfill & {} \hfill & {} \hfill & {} \hfill & {} \hfill & {} \hfill & {} \hfill \\ \end{array} } \right]\left[ {\begin{array}{*{20}l} {x_{0} } \hfill \\ {x_{1} } \hfill \\ {x_{2} } \hfill \\ \vdots \hfill \\ {} \hfill \\ \end{array} } \right]\left( t \right)$$

where $f_{i}$ is the per-capita fertility and $\phi_{i}$ is the survival probability (per cycle) of an age class i. This is the correct form for mosquitoes under the commonly-made assumption of no senescence (age-independent survival).

Under certain conditions in which there is an oldest age class $m$ that does not survive ($\phi_{m} = 0$; ‛Leslie matrix’) or when the oldest age class represents all animals of this age and older with the same survival probability (see [16]; ‛Usher matrix’) the infinite matrix in (Eqn. 2) can be treated as finite as follows:

3 $$\left[ {\begin{array}{*{20}l} {x_{0} } \hfill \\ {x_{1} } \hfill \\ {x_{2} } \hfill \\ \vdots \hfill \\ {x_{m} } \hfill \\ {} \hfill \\ \end{array} } \right]\left( {t + 1} \right) = \left[ {\begin{array}{*{20}l} {f_{0} } \hfill & {f_{1} } \hfill & {f_{2} } \hfill & {f_{3} } \hfill & \ldots \hfill & \ldots \hfill & {f_{m} } \hfill \\ {\phi_{0} } \hfill & 0 \hfill & 0 \hfill & 0 \hfill & \ldots \hfill & \ldots \hfill & 0 \hfill \\ 0 \hfill & {\phi_{1} } \hfill & 0 \hfill & 0 \hfill & \ldots \hfill & \ldots \hfill & 0 \hfill \\ \vdots \hfill & \vdots \hfill & \vdots \hfill & \vdots \hfill & \ddots \hfill & 0 \hfill & 0 \hfill \\ 0 \hfill & 0 \hfill & 0 \hfill & 0 \hfill & \ldots \hfill & {\phi_{m - 1} } \hfill & {\phi_{m} } \hfill \\ {} \hfill & {} \hfill & {} \hfill & {} \hfill & {} \hfill & {} \hfill & {} \hfill \\ \end{array} } \right]\left[ {\begin{array}{*{20}l} {x_{0} } \hfill \\ {x_{1} } \hfill \\ {x_{2} } \hfill \\ \vdots \hfill \\ {x_{m} } \hfill \\ {} \hfill \\ \end{array} } \right]\left( t \right)$$

In both cases, certain stable population theory results apply with only weak assumptions [16], most notably that the long-term population structure is attained irrespective of the initial conditions (it is ‛ergodic’ [17]). The number of individuals in age class i at this stable age structure is given by:

4 $$x_{i} = x_{0} \lambda^{ - i} \prod\limits_{k = 0}^{i - 1} {\phi_{k} }$$

where $\lambda$ is the population growth rate.

Conventional mosquito models commonly suppose equal (age-independent) survival probabilities ($\phi$, say) of the age classes $\phi_{0} = \phi_{1} = \ldots \phi_{m} = \phi$, and age-independent fertility $f_{0} = f_{1} = \ldots f_{m} = f$. A further common assumption is that mosquito age-classes are of the same duration (most likely the oviposition or gonotrophic cycle length). At the stable age distribution the number in age class i reduces to [17]:

5 $$x_{i} = x_{0} \left( {\frac{\phi }{\lambda }} \right)^{i}$$

So if furthermore the population is stationary ($\lambda = 1)$ then the relationship between adjacent age classes is given by:

6 $$\frac{{x_{i} }}{{x_{i - 1} }} = \phi$$

Many mosquito studies have used this result, at least implicitly.

***Stage-structured matrix*** An alternative formulation is a stage-structured model [17], where individuals are classified by a life-stage or size, rather than an age. Unlike a Leslie model, stage-classified models allow ‛self-loops’ where a state ‛transition’ may lead to itself. Physiological markers of age that may be used in the mosquito literature are described by Silver [11]. The most frequent marker of stage that has been utilised is the egg-laying condition of the female mosquito, most simply (and most commonly) whether or not the mosquito has previously laid (is parous) or not laid eggs (is nulliparous). For a nulliparous/parous model:

7 $$\left[ {\begin{array}{*{20}l} {x_{0} } \hfill \\ {x_{p} } \hfill \\ {} \hfill \\ \end{array} } \right]\left( {t + 1} \right) = \left[ {\begin{array}{*{20}l} 0 \hfill & f \hfill \\ \phi \hfill & \phi \hfill \\ {} \hfill & {} \hfill \\ \end{array} } \right]\left[ {\begin{array}{*{20}l} {x_{0} } \hfill \\ {x_{p} } \hfill \\ {} \hfill \\ \end{array} } \right]\left( t \right)$$

For a population at equilibrium we have $\frac{{x_{p} }}{{x_{0} }} = \frac{\phi }{1 - \phi }$ (cf. Eqn. 6) or

8 $$\phi = \frac{{x_{p} }}{{x_{0} + x_{p} }}$$

A stage-structured model may not have the ‛ergodic’ property, i.e. its long-term state may depend on initial conditions.

***Disease state matrix*** After initial infection the malarial parasite develops over the period of the ‛extrinsic incubation period’ (EIP) to cause the mosquito host to become infected with sporozoites. The infectivity of the mosquito might be seen as a crude marker of age, or explicitly characterised by its disease-carrying state. For the latter, and to demonstrate the connection with other approaches above, we write the process in a discrete-time matrix form (compare with earlier Eqns. 2 and 7 where mosquitoes were categorised by their age or parity):

9 $$\left[ {\begin{array}{*{20}l} {x_{S} } \hfill \\ {x_{E} } \hfill \\ {x_{I} } \hfill \\ {} \hfill \\ \end{array} } \right]\left( {t + n} \right) = \left[ {\begin{array}{*{20}l} {f + \left( {1 - \omega } \right)\xi } \hfill & f \hfill & f \hfill \\ {\omega \xi } \hfill & {\left( {1 - \gamma } \right)\xi } \hfill & 0 \hfill \\ 0 \hfill & {\gamma \xi } \hfill & \xi \hfill \\ {} \hfill & {} \hfill & {} \hfill \\ \end{array} } \right]\left[ {\begin{array}{*{20}l} {x_{S} } \hfill \\ {x_{E} } \hfill \\ {x_{I} } \hfill \\ {} \hfill \\ \end{array} } \right]\left( t \right)$$

where $S$,$E$,$I$ denote susceptible, exposed (infected but not infectious) and infectious mosquitoes; $\omega$ is the probability of infection (susceptible mosquito transitions to infected); $\gamma$ is the probability an infected mosquitoes transitions to an infectious state; $\xi$ is the probability of a mosquito surviving the EIP, assumed the same for all classes, and $n$ is the EIP in days.

Note that $\xi$ can be written in terms of daily survival as $p^{n}$.

We stress that Eqn. 9 is highly simplified: the probability of infection term $\omega$ is a function of other parameters including in particular the number of infectious hosts such as humans. The equation is clearly part of a wider disease system which includes vertebrate host dynamics as well. A much fuller form of this discrete-time system has been studied [18].

We can examine the system when by assumption it is at equilibrium so $$\underset{\raise0.3em\hbox{$\smash{\scriptscriptstyle-}$}}{x} \left( {t + n} \right) = \underset{\raise0.3em\hbox{$\smash{\scriptscriptstyle-}$}}{x} \left( t \right) = \underset{\raise0.3em\hbox{$\smash{\scriptscriptstyle-}$}}{x}^{ *}$$, say. If transition from infected to infectious is certain ($$\gamma = 1$$) then the third row of Eqn. 9 yields that $$\xi \left( {x_{E}^{ *} + x_{I}^{ *} } \right) = x_{I}^{ *}$$ so:

10 $$\xi = \frac{{x_{I}^{ *} }}{{\left( {x_{E}^{ *} + x_{I}^{ *} } \right)}}$$

The right hand side can be written equivalently and in a more familiar form as $s/Y$. Here, $s$ is the proportion of infectious (the proportion of mosquitoes containing sporozoites in the salivary glands) and $Y$ is the proportion of infected mosquitoes.

It is much more common in the literature to use continuous-time form of dynamical equations to describe the disease state system, rather than discrete-time form. Using the former, various authors provide expressions for the sporozoite rate $s$ which [2] writes as :

$$\frac{acX}{g + acX}{ \exp }\left( { - gn} \right)$$

where $a$ is the biting rate, $g$ is the continuous mortality rate, $c$ is the probability an uninfected mosquito becomes infected after biting an infectious human and $X$ is the proportion of infected humans. The left hand term is the proportion of infected mosquitoes ($Y$) and the right hand term is the probability of surviving the EIP ($\xi$), so that again $s = Y\xi$.

#### Estimation

This section elaborates on the estimation process for the population models above. We will abbreviate some of the estimation methods (LRH, LRV, JS, or FF) as explained further below.

***Proportion parous*** A cross-sectional sample of the stage-structure with an assumption of stability gives an estimate of $\phi$ (Eqn. 8). Another way of looking at this that is often used may go back to [19]. Suppose all age classes are sampled representatively and survival is constant. Let $f$ be the number of cycles before which the mosquito begins to lay eggs, so that the expected number nulliparous is $x_{n} = \sum\nolimits_{0}^{f} {x_{i} }$ and the expected numbers in older, now parous, age classes are $x_{f + 1} ,x_{f + 2} , \ldots$. Then the proportion nulliparous is

11 $$\frac{{x_{n} }}{{x_{n} + x_{f + 1} + x_{f + 2} + \cdots }} = \frac{{x_{n} }}{{x_{n} \left( {1 + \phi + \phi^{2} + \cdots } \right)}} = \left( {1 - \phi } \right)$$

In this way the proportion parous is an estimate of the survival rate over a cycle.

It is also possible to use a time series approach [20]. Assuming the presence of sampling error in time series of estimates of parous $X_{p} \left( 1 \right),X_{p} \left( 2 \right), \ldots$ and nulliparous mosquitoes $X_{0} \left( 1 \right),X_{0} \left( 2 \right), \ldots$, then $X_{p} \left( {t + 1} \right) = \phi \left[ {X_{0} \left( t \right) + X_{p} \left( t \right)} \right] + \varepsilon$ can be solved by least squares for an estimate of $\phi$.

***Regression approach (LRV)*** Starting with the relation $x_{i} = x_{0} \phi^{i} ,i = 1, \ldots ,m$ when the population is stationary (see Eqn. 5) and taking logarithms of the expected values, ${ \log }\left( {E\left[ {x_{i} } \right]} \right) \approx E\left[ {{ \log }\left( {x_{i} } \right)} \right] = { \log }\left( {x_{0} } \right) + i.{ \log }\left( \phi \right)$. With normally-distributed errors ${ \log }\left( {x_{i} } \right) \approx { \log }\left( {x_{0} } \right) + i.{ \log }\left( \phi \right) + \varepsilon$ which can be solved by regression, and the estimated coefficient can be back-transformed for an estimate of $\phi$. The method is outlined for example by [21].

***Parasitological estimate*** We reinterpret a concise argument [22] to estimate $\xi$ (and therefore $p$) as follows. A sample of wild-caught mosquitoes at $t$ is assessed for the proportion infectious, to give the ‛immediate sporozoite rate’. Another sample from the same population is kept alive for the duration of the EIP ($n$), and also assessed for the proportion infectious, now at $t + n$ (the ‛delayed sporozoite rate’). We assume there are no losses since the mosquitoes are protected from natural sources of mortality after $t$, and by assumption there is no senescence. We further assume that all infected (but not yet infectious) mosquitoes pass to infectious by the $t + n$ sample. The mosquitoes infectious at $t + n$ is made up of those infectious at $t$, plus any infected at $t$ and becoming infectious over the EIP : $x_{I} \left( {t + n} \right) = x_{I} \left( t \right) + x_{E} \left( t \right)$. So an estimate of the number infected but not infectious at $t$ is $\hat{x}_{E} \left( t \right) = x_{I} \left( {t + n} \right) - x_{I} \left( t \right)$. The ratio of infectious: infected at all is then

12 $$\frac{{x_{I} \left( t \right)}}{{x_{I} \left( t \right) + \hat{x}_{E} \left( t \right)}} = \frac{{x_{I} \left( t \right)}}{{x_{I} \left( {t + n} \right)}}$$

This ratio can be related to survival: as shown above (Eqn. 10), the ratio ($s/Y$) is the probability of surviving the EIP ($\xi$), from which $p$ or $\phi$ can be found. Saul et al. [23] and followers modified this approach to estimate survival over a feeding cycle under ‛natural’ conditions, using parameters more practicable to estimate, particularly the proportions infected in a biting catch and infected in a resting (fed) catch.

Macdonald [24] presented a number of heuristic estimates surmised from other authors’ infection data, essentially by solving an analogue of Eqn. 10.

### Horizontal methods

#### Mark-recapture

Cohorts of mosquitoes marked in some way are followed up over time and the times of recovery, and perhaps rerelease, are analysed. The marked population is under the control of the investigator including the times of entry of newly marked mosquitoes. Mosquitoes may be killed on capture, or re-released, with or without new marks. A model for the survival of released mosquitoes over time and their probabilities of recapture is used to estimate survival parameters. In contrast to vertical methods, no assumptions are made that the population has attained equilibrium or a stable age-structure.

Most mark-recapture (MR) mosquito studies are single-release experiments. A size $m_{0}$ sample of marked mosquitoes is released and the numbers recaptured at future times are recorded. A minority of MR experiments are multiple release. At a recapture occasion, more mosquitoes are released. These may be mosquitoes marked previously, or newly marked.

#### Estimation

***Single release*** Let $m_{0}$ be the number of mosquitoes marked at time 0 and $m_{1} ,m_{2} ,m_{3} , \ldots$ be the numbers of those recaptured at later times. Let $\pi$ be the (constant) probability of recapture on any occasion. The expected number recaptured at time k is (see [25] but with a different notation):

13 $$E\left( {m_{k} } \right) = m_{0} \phi^{k} \pi (1 - \pi )^{k - 1}$$

By far the most common approach to estimating $\phi$ is to express Eqn. 13 as a regression equation from which $\phi$ may be estimated [25]:

14 $$E\left( {log\left( {m_{k} + 1} \right)} \right) \approx log\left( {m_{0} \pi } \right) + \left( {k - 1} \right)log\left( {1 - \pi } \right) + k.log\left( \phi \right)$$

with a unit added to $m_{k}$ to ensure computability if zero counts arise [25]. A regression without the middle term might be used (see e.g. [21]), which might be satisfactory if mosquitoes are re-released, or if $\pi$ is small.

***Multiple release*** A number of methods exist which, though often aimed at estimates of abundance, may also estimate survival. These are relatively complex and we refer readers elsewhere for full details, e.g. [6, 26]. We briefly cover the three we encountered:

(i) The Fisher-Ford (FF) method’s primary function is to estimate population size. Nevertheless it contains an associated estimate of survival that has been utilised by a few authors. The method assumes time-independent survival, and uses the average observed survival time of marked individuals that survive to recapture, and the expected average survival times for those released given $\phi$ [26] . For the simpler case of a single recapture time $k$ capturing in total $m_{k}$ previously marked mosquitoes (see [26] for further extension to multiple recaptures), and with $r_{j}$ denoting the number of these released j days before, the *observed* average survival time is:

$$\frac{{\sum\nolimits_{j} {r_{j} j} }}{{m_{k} }}$$

Denoting by $a_{j}$ the number newly released $j$ days before the sampling time $k$, the *expected* average survival time (of released mosquitoes surviving to time $k$) is:

$$\frac{{\sum\nolimits_{j} {a_{j} \phi^{j} j} }}{{\sum\nolimits_{j} {a_{j} \phi^{j} } }}$$

An estimate of $\phi$ is fitted that equates observed and expected average survival time.

(ii) The Jolly-Seber (JS) method uses multiple releases and recaptures to estimate (time-dependent) survival (and other parameters, notably abundance) and is in common use by ecologists [6, 26]. The essence of the method is to estimate survival from estimates of the marked population sizes $M_{t}$ and $M_{t + 1}$ at adjacent times i.e. $\hat{\phi } = \frac{{M_{t + 1} }}{{M_{t} }}$. Estimates of $M_{t}$ are obtained by assuming the future recapture rates of already marked animals *not* caught at time $t$ is the same as the future recapture rate of the marked animals released at time $t$. Under the basic model, survival rate may vary with time but it can be modified to allow constraints (e.g. time-independent survival) and doing so can improve the precision of the estimates. In mosquito studies the JS method is usually applied in full, though the model contains a component (the ‘Cormack-Jolly-Seber’ model) that is sufficient for estimating survival.

(iii) Saul [27] developed their own estimates which involve algebraic solutions to MR equations. The method supposes time-independent survival.

### Other estimation methods

Other methods were uncommon and we do no more than mention them. These included the rate of population decline under conditions of zero recruitment [28, 29]; the ‛Manly-Parr’ method [26], applied by [30] (though no survival estimate was given for this method); and informal approaches (e.g. fitting a survival curve graphically [9]).

### Search and meta-analysis

In order to capture estimates of survival the authors developed a systematic search strategy and ran it in the following databases: PubMed (National Library of Medicine), Global Health (OvidSP), Web of Science Core Collection (Clarivate Analytics), Environment Complete (EBSCOhost) and Scopus. The searches were carried out in November 2017 with no date or language limitations. Scoping indicated that Web of Science would give the most relevant results so used a broader strategy than the others. Web of Science search strategy: (Mosquito* or anophel*)TI AND (surviv* OR longevity OR mortality OR lifecycle* or “life cycle*”)TS. Environment Complete, Global Health, Scopus and PubMED search strategy: (Mosquito* or anophel*)TI AND (surviv* OR longevity OR mortality OR lifecycle* or “life cycle*)TI.

Exclusions were then made of unpublished or non English-language studies, interventions that might affect survival e.g. insecticide; studies without natural sources of mortality (laboratory studies) except for studies using the ‛immediate: delayed sporozoite rate’ (see above), which only supposes no mortality at future timepoints, beyond the timepoint of the estimate; studies reanalysing data with an age-dependent model; methodological/simulation studies; review papers or other duplicate estimates; and studies not providing estimates. Where only a parous proportion was supplied we treated that as an estimate of probability of cycle survival.

We planned to carry out meta-analyses with studies weighted by inverse-variance weighting. However most vertical studies and single-release mark-recapture did not provide variance estimates or related metrics (confidence intervals etc). We therefore carried out unweighted meta-analyses. Daily survival rates were transformed to log(odds) prior to meta-analysis and then back-transformed for presentation. Analysis was carried out in R 3.5 with the package *metafor*.

## Results

A total of 5124 records were identified from the database searching which was reduced to 3529 once the duplicates had been removed. These records were screened at title and (when available) abstract by two of the authors (JM or GO), at which point it was decided to include only English language records. After applying exclusion criteria, 84 publication records were selected: of these 55 had been found by database searching, and 29 were by supplementary searching or in the reference collection of one of the authors (JM). Articles found by supplementary searching or author collection were mostly early (25 of 29 were published prior to 2000) and less likely to be found in search databases. The final inclusions (listed in Additional file 1: Text S1) contained 174 species-specific estimates of the common literature metrics of daily survival ($p$) and cycle survival ($\phi$) for the synthesis.

**Fig. 1:**
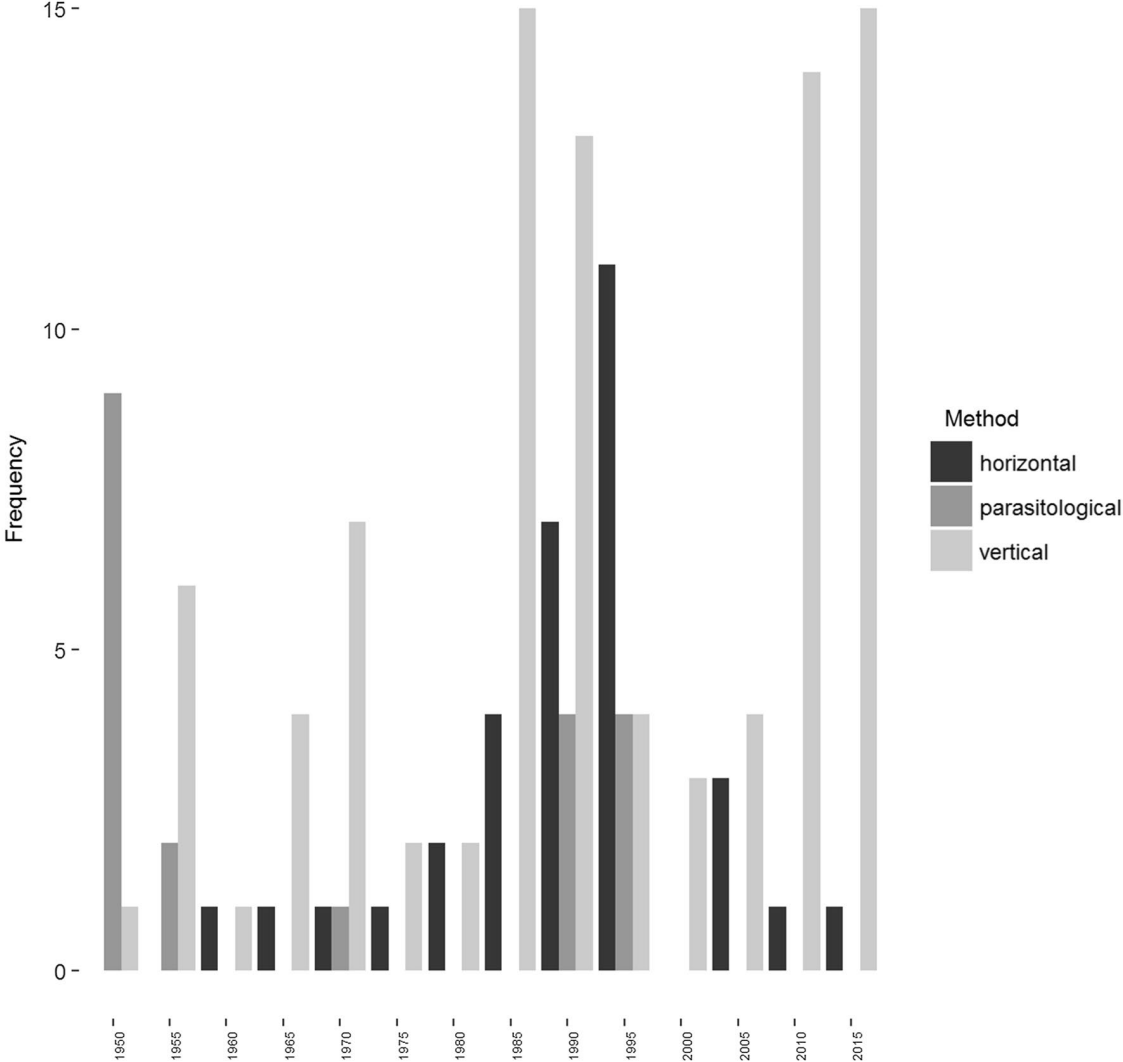
Frequency of published estimates of daily survival probability over time (in half-decades), by method (vertical, horizontal or parasitological)

The publication of survival estimates categorised by their broad methodology is shown in Fig. 1. Parasitological methods were prominent in the 1950s [24] and in the 1990s with the advent of the approach of Saul et al. [23]. Vertical methods, particularly those based on parity, have been in common usage throughout the period covered. Horizontal methods are also in regular use though with something of a peak in the 1980–1990s. A more detailed indication of the frequency of analysis methods is shown in Table 1.

**Table 1:** Frequency of estimates by daily or cycle survival and analysis method with associated explanatory references

Methodological approach		Reference	Daily survival (p)	Cycle survival (Ф)
Horizontal	Regression (LRH)	Milby and Reisen [21]	29	5
	Mark-recapture (FF)	Begon [26]	3	0
	Mark-recapture (JS)	McRea et al. [6]	1	0
	Mark-recapture	Saul et al. [27]	0	2
Vertical	Regression (LRV)	Milby and Reisen [21]	16	7
	Parous rate	Davidson [19]	64	71
	Parous rate by time-series	Birley et al. [20]	2	7
Parasitological	Immediate:delayed	Davidson and Draper [22]	5	0
	Infection rate	Saul et al. [23]	0	8
	Infection rate	MacDonald [24]	7	0

**Fig. 2:**
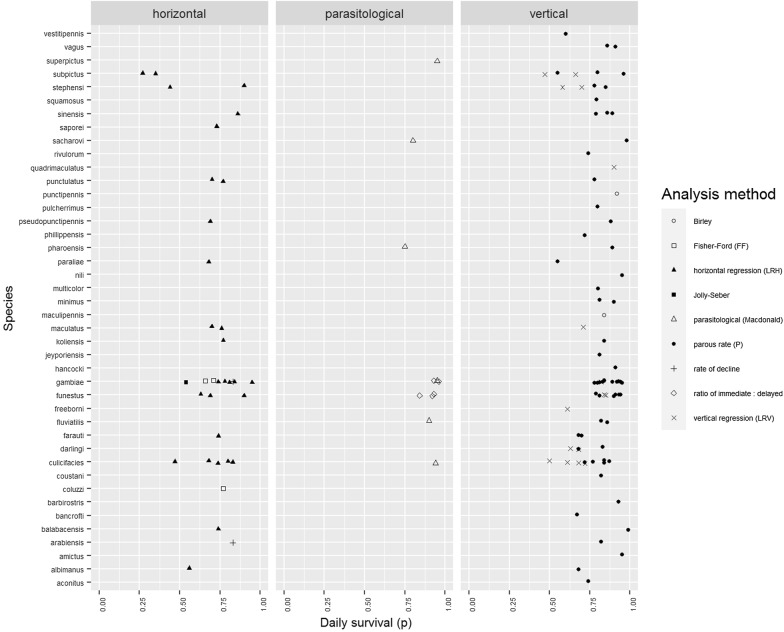
Probability of daily survival by *Anopheles* species by method (vertical, horizontal or parasitological) and analysis

**Fig. 3:**
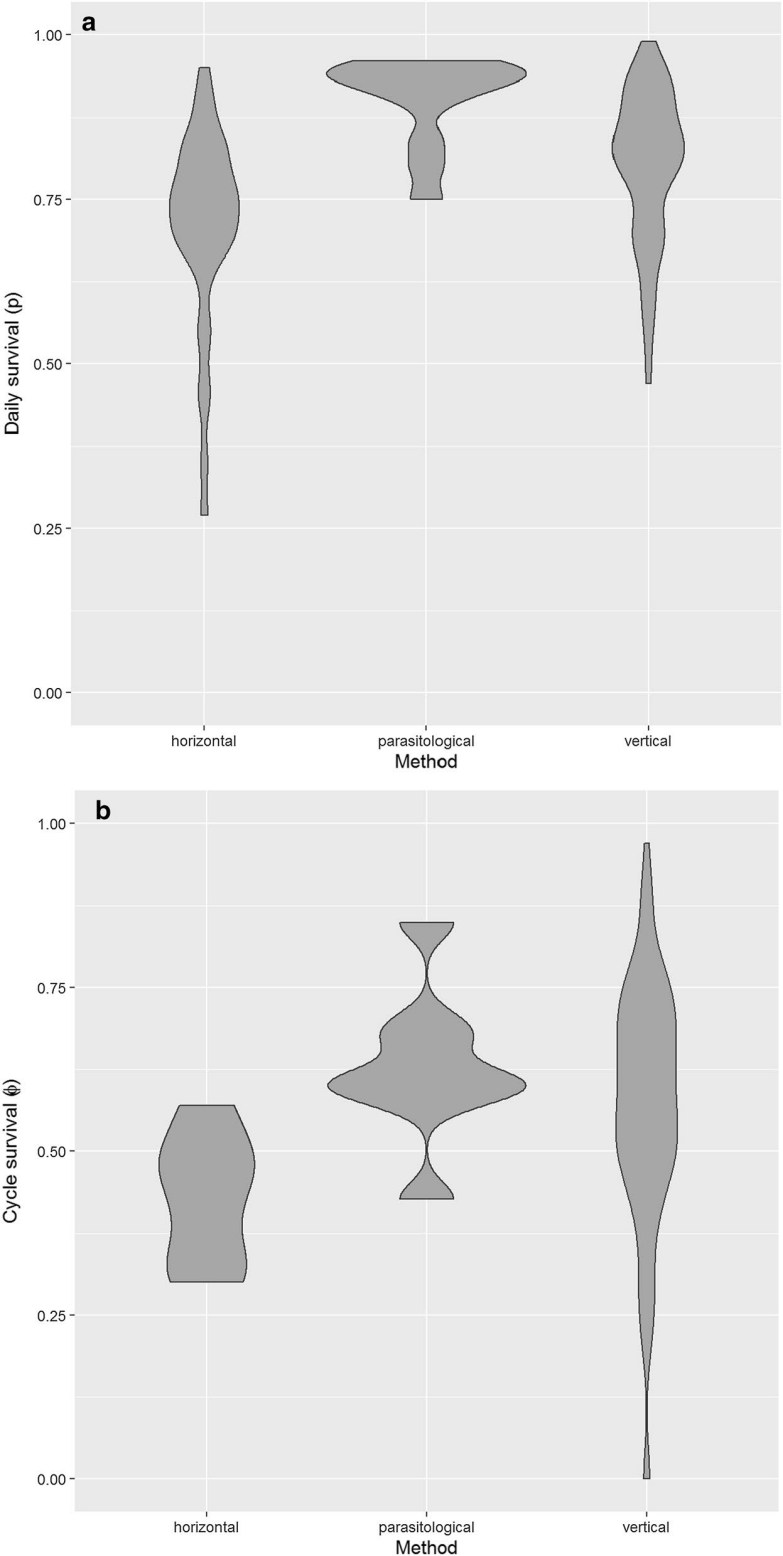
Violin plot of probability of daily survival (**a**) and cycle survival (**b**). These show the (smoothed) density of estimates by method (vertical, horizontal or parasitological)

Daily survival ($p$) estimates are shown in Fig. 2 categorised by their broad methodology. Estimates of the centre of the distribution of $p$ from meta-analyses are shown in Table 2 and a violin plot summarising the density of the survival estimates is shown in Fig. 3a. Horizontal estimates of the centre are lower [$p$= 0.73 (95% confidence interval, CI: 0.66–0.79), expected lifetime of 3.2 days] than for vertical methods [$p$= 0.83 (95% CI: 0.80–0.86), expected lifetime of 5.7 days]. Parasitological estimates are noticeably high with a central estimate of $p$= 0.92 (95% CI: 0.86–0.95, expected lifetime of 8.6 days). The analagous figures for cycle survival ($\phi$) are shown in Fig. 3b and Additional file 2: Figure S1.

**Table 2:** Estimates of probability of daily survival (p) with 95% CI under unweighted meta-analysis

Methodological approach		p (95% CI)
Horizontal	Pooled	0.73 (0.66–0.79)
	Fisher-Ford	0.72 (0.45–0.89)
	Jolly-Seber	0.54 (0.14–0.89)
	Horizontal regression	0.73 (0.65–0.80)
Parasitological	Pooled	0.92 (0.86–0.95)
	Infection rate^a^	0.91 (0.83–0.96)
	Immediate:delayed	0.92 (0.83–0.97)
Vertical	Pooled	0.83 (0.80–0.86)
	Parous rate	0.85 (0.82–0.88)
	Vertical regression	0.70 (0.58–0.79)

^a^Method of MacDonald [24]

*Abbreviation*: CI, confidence interval

**Fig. 4:**
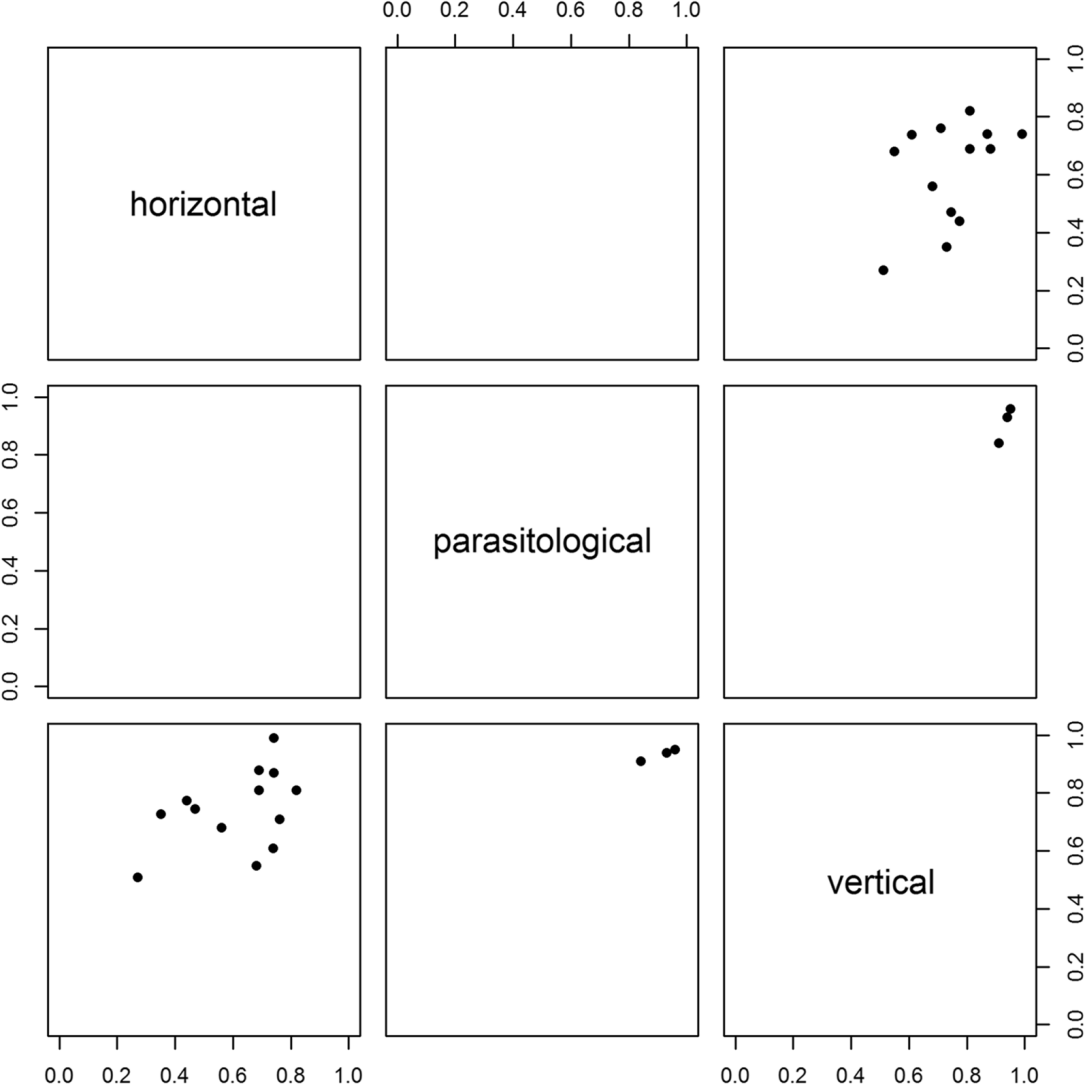
Comparison of estimates of probability of daily survival between methods (vertical, horizontal or parasitological), for those studies that used more than one such method simultaneously

Results where multiple methods have been used to make simultaneous estimates are shown in Fig. 4. Some correlation is observed but it is not strong.

## Discussion

We believe that the results given here supply a systematic overview of anopheline survival under wild conditions that has been lacking, particularly in relation to the methods adopted by researchers. Our results clearly demonstrate the dependency of estimates on the methods used. Many authors are and were aware of potential biases when estimating survival and the issues were summarised by Gillies [7], but there does not appear to be any concensus about the preferred approach and research continues using all these methods (Fig. 1). Authors are only occasionally explicit about the reasons for their choice of method (e.g. [31]).

The lower estimates of $p$ provided by ‛horizontal’ methods (Figs. 3 and 4) were anticipated (e.g. [7]): this is an estimate of ‛apparent’ survival (the probability of surviving and not emigrating from the study area) and that is its chief disadvantage. That aside, with the longitudinal method a high degree of control is possible and can incorporate environmental covariates and time-dependency [6]. Where assumptions are made (e.g. random mixing of the population, effect on mortality of marking) these can be assessed. The effect of typical characteristics of mosquito mark-recapture estimators can be examined theoretically or by simulation (these characteristics include large numbers of releases in batches and a low recapture rate generally 5–10% or less).

While vertical methods can provide estimates of true not apparent survival (i.e. where mortality is not confounded with emigration) they make assumptions about stationarity or stability. Some authors take care to provide at least partial evidence of stability [32] but this is unusual. It seems likely to us that vertical methods of survival estimation will be underpinned by mathematical rather than empirical arguments.

We have attempted to illustrate the connections between vertical methods using a mathematically simple discrete-time matrix framework. For example: Birley’s method for estimation is developed from the parous/nulliparous stage-classified model (Eqn. 7), and the parasitological approach makes use of a disease stage-classified model at equilibrium (Eqn. 9). Furthermore, assumptions have been illustrated in greater detail. For example Davidson & Draper’s method [19] makes assumption about the certainty of transition from infected to infectious after the EIP ($\gamma = 1$ in Eqn. 9).

The parasitological method [22] (‛immediate: delayed sporozoite rate’; Eqn. 12) tends to estimate a high survival probability centred at $p$= 0.92 (Figs. 2 and 3a, Table 2). One potential problem here is the assumption that an infected mosquito will proceed to an infectious state by the end of the EIP, as there is evidence that some infections may be cleared by mosquitoes [23]. If the number infectious counted in the sample at the end of the EIP is smaller because infected mosquitoes do not in fact all transition to an infectious state, then the probability of surviving the EIP $\xi$ by Eqn. (10) will be overestimated.

Within the vertical methods we found a lower survival estimate by regression over age groups ($p$ = 0.70) than by the parous rate ($p$ = 0.85). This pattern was previously found and commented on by Gillies & Wilkes [8], who pointed to the undersampling of nulliparous mosquitoes in house catches as potentially problematic (the nullipars were excluded from their regression estimate for this reason).

There is a multitude of further issues that are large and involved and could not all be feasibly discussed here. We give two examples to illustrate. First, in estimating daily survival a figure for the ‛gonotrophic’ cycle is frequently used. Some authors treat the gonotrophic cycle (between feeds) as equivalent in duration to the oviposition cycle (between clutches). Operationally the duration of the gonotrophic or oviposition cycle is used in the same way, to transform survival probability over a cycle to daily survival probability. This equivalence can be undermined because for example “the first gonotrophic cycle is an atypical one. For one thing it may involve more than one blood meal …” [7]. Secondly, a (long-standing) assumption of age-independent mortality underlies many of the estimation methods that have been used. Its validity is sometimes disputed [10, 14] and alternative analyses with age-dependence are possible (e.g. fitting of alternative parametric curves [10]). Age-dependent survival estimates do not map to the age-independent $p$ targetted in the majority of studies and synthesised here, and this important (if unusual) alternative is excluded from our study.

The search strategy we employed should capture most direct survival estimates but information from indirect reporting will not be found. For example, Lines et al. [33] reported survival rate estimates but the study was not discovered by our search strategy.

In principle, a reanalysis of published data could be carried out to apply alternative or preferable models [10, 25], or increase the available sample size (for example where a reported age-structure, e.g. results in [34], might be further analysed to give a survival estimate). Importantly, we found that the typical estimate of survival did not supply an estimate of its own uncertainty, and this could be rectified to a limited extent by reanalysis. For example, publications using regression (horizontal or vertical) often provide summary data suitable for reanalysis, but on the other hand the information required for Jolly-Seber reanalysis is often unavailable. We are not aware of error estimates for Fisher-Ford nor for parasitological analyses except for Saul’s approach (appendix of [35]). The proportion parous might be treated as a binomial variable though doing so would involve assumptions (e.g. the age-structure is precisely known, sampling is representative, there were no clustering effects).

The estimates of daily probability presented here were carried out with an unweighted meta-analysis: the preferred approach of weighting by inverse variance was not possible as we lacked study-level indications of uncertainty in very many cases, a common situation in meta-analysis [36]. Had study-level uncertainty estimates been available then (i) a separation of between-study and within-study variation could have been made leading to estimates of heterogeneity; and (ii) a more precise estimate of $p$ obtained. On the other hand, point estimates from unweighted meta-analyses can be unbiased [36] and in that sense reliable.

The between-method variation in estimates of $p$ suggests there is considerable methodological bias (such as that identified by Buonaccorsi et al. [25]) to go along with known differences in the target of estimation (survival *versus* apparent survival). The extent of within-method variation (Figs. 2 and 3) is, in contrast, not surprising: $p$ is determined by many further factors including species-specific effects, environmental factors and differences in study site characteristics (e.g. method of baiting or trapping, area of site).

There are many important environmental factors affecting mosquito survival including temperature, humidity, sources of predation and availability of nutrition. These have not been investigated in the present work, which focusses on differences arising from choice of methodology. Such information is largely absent from the included studies (in principle climatic information can be imputed [13] though there is a limit to its resolution). There was no indication in any of the studies included that the choice of method was made on the basis of environmental factors, which implies that the reported distributional centres when separated by method are largely independent of them. These factors do contribute to the variation in estimates (heterogeneity) seen in Figs. 2 and 3.

## Conclusions

We carried out a systematic search for estimates of anopheline mosquito survival in ‛natural’ conditions, with particular emphasis on the methods used and brief explanations of them. We estimated (with CIs) the average of the commonly used metric, daily survival ($p$), by method. The choice of method strongly influences the estimate and we quantified the differences. The between-method differences reflect methodological biases that should be taken into account when utilising the estimates.

## Supplementary information

**Additional file 1: Text S1.** List of included papers providing survival estimates.

**Additional file 2: Figure S1:** Probability of cycle survival by *Anopheles* species and by method (vertical, horizontal or parasitological) and analysis.

## Data Availability

The datasets analysed during the present study are available from the corresponding author upon reasonable request.

## Abbreviations

EIP: Extrinsic incubation period
JS: Jolly-Seber
FF: Fisher-Ford
LRH: Horizontal linear regression
LRV: Vertical linear regression
MR: Mark-recapture
