# Supplementary material for: An overview of malarial Anopheles mosquito survival estimates in relation to methodology
Source: Parasit Vectors. 2020 May 7;13:233. doi: 10.1186/s13071-020-04092-4 (PMC7206813; doi:10.1186/s13071-020-04092-4)
Supplement: Supplementary file 1 — Additional file 1: Text S1. List of included papers providing survival estimates. [file 13071_2020_4092_MOESM1_ESM.docx]

**Additional file 1: Text S1.** List of included papers providing survival estimates.

Afifi SE, Spencer M, Hudson PB, Tavil NW. Biting prevalence and malaria transmission patterns in the Anopheles punctulatus complex (Diptera: Culicidae) in Papua New Guinea. The Australian journal of experimental biology and medical science. 1980;58(1):1-17.

Aniedu I, Mutinga MJ, Mutero CM. Age Composition and Survival Rate of Anopheles-Gambiae Giles Complex (Dipt, Culicidae) in Baringo District, Kenya. Journal of Applied Entomology-Zeitschrift Fur Angewandte Entomologie. 1989;107(4):387-94.

Arredondo-Jimenez JI, Rodriguez MH, Washino RK. Gonotrophic cycle and survivorship of Anopheles vestitipennis (Diptera : Culicidae) in two different ecological areas of Southern Mexico. Journal of Medical Entomology. 1998;35(6):937-42.

Bugoro H, Cooper RD, Butafa C, Iro'ofa C, Mackenzie DO, Chen CC, et al. Bionomics of the malaria vector Anopheles farauti in Temotu Province, Solomon Islands: issues for malaria elimination. Malaria Journal. 2011;10.

Burkot TR, Graves PM, Paru R, Battistutta D, Barnes A, Saul A. Variations in Malaria Transmission Rates Are Not Related to Anopheline Survivorship Per Feeding Cycle. American Journal of Tropical Medicine and Hygiene. 1990;43(4):321-7.

Catangui FP. Studies on the gonotrophic cycle of Anopheles minimus flavirostris and the application of physiological age grading technique on the same species. Southeast Asian J Trop Med Public Health. 1971;2(3):384-92.

Charlwood JD. OBSERVATIONS ON THE BIONOMICS OF ANOPHELES-DARLINGI ROOT (DIPTERA, CULICIDAE) FROM BRAZIL. Bull Entomol Res. 1980;70(4):685-92.

Charlwood JD. Survival Rate Variation of Anopheles-Farauti (Diptera, Culicidae) between Neighboring Villages in Coastal Papua-New-Guinea. Journal of Medical Entomology. 1986;23(4):361-5.

Charlwood JD. Vectorial capacity, species diversity and population cycles of anopheline mosquitoes (Diptera: Culicidae) from indoor light-trap collections in a house in southeastern Tanzania. African Entomology. 1997;5(1):93-101.

Charlwood JD, Alecrim WA. CAPTURE-RECAPTURE STUDIES WITH THE SOUTH-AMERICAN MALARIA VECTOR ANOPHELES-DARLINGI, ROOT. ANN TROP MED PARASIT. 1989;83(6):569-76.

Charlwood JD, Bryan JH. A MARK RECAPTURE EXPERIMENT WITH THE FILARIASIS VECTOR ANOPHELES-PUNCTULATUS IN PAPUA-NEW-GUINEA. ANN TROP MED PARASIT. 1987;81(4):429-36.

Charlwood JD, Graves PM, Birley MH. CAPTURE-RECAPTURE STUDIES WITH MOSQUITOS OF THE GROUP OF ANOPHELES-PUNCTULATUS DONITZ (DIPTERA, CULICIDAE) FROM PAPUA-NEW-GUINEA. Bull Entomol Res. 1986;76(2):211-27.

Charlwood JD, Kihonda J, Sama S, Billingsley PF, Hadji H, Verhave JP, et al. The Rise and Fall of Anopheles Arabiensis (Diptera, Culicidae) in a Tanzanian Village. Bull Entomol Res. 1995;85(1):37-44.

Charlwood JD, Nenhep S, Sovannaroth S, Morgan JC, Hemingway J, Chitnis N, et al. 'Nature or nurture': survival rate, oviposition interval, and possible gonotrophic discordance among South East Asian anophelines. Malaria Journal. 2016;15.

Charlwood JD, Smith T, Billingsley PF, Takken W, Lyimo EOK, Meuwissen J. Survival and infection probabilities of anthropophagic anophelines from an area of high prevalence of Plasmodium falciparum in humans. Bull Entomol Res. 1997;87(5):445-53.

Charlwood JD, TomÁS EV, Egyir-Yawson A, Kampango AA, Pitts RJ. Feeding frequency and survival of Anopheles gambiae in a rice-growing area in Ghana. Medical & Veterinary Entomology. 2012;26(3):263-70.

Charlwood JD, Wilkes TJ. STUDIES ON THE AGE-COMPOSITION OF SAMPLES OF ANOPHELES-DARLINGI ROOT (DIPTERA, CULICIDAE) IN BRAZIL. Bull Entomol Res. 1979;69(2):337-42.

Chiang GL, Loong KP, Chan ST, Eng KL, Yap HH. Capture-recapture studies with Anopheles maculatus Theobald (Diptera: Culicidae) the vector of malaria in peninsular Malaysia. Southeast Asian J Trop Med Public Health. 1991;22(4):643-7.

Costantini C, Li SG, DellaTorre A, Sagnon N, Coluzzi M, Taylor CE. Density, survival and dispersal of Anopheles gambiae complex mosquitoes in a West African Sudan savanna village. Med Vet Entomol. 1996;10(3):203-19.

Curtis CF, Rawlings P. Preliminary-Study of Dispersal and Survival of Anopheles-Culicifacies in Relation to the Possibility of Inhibiting the Spread of Insecticide Resistance. Ecol Entomol. 1980;5(1):11-7.

Dagoro H, Paru R, Holmes PR, Birley MH, Charlwood JD. Assessing survival rates of Anopheles farauti (Diptera: Culicidae) from Papua New Guinea. J Anim Ecol. 1985;54(3):1003.

Davidson G. Estimation of the Survival-Rate of Anopheline Mosquitoes in Nature. Nature. 1954;174(4434):792-3.

Davidson G. Further studies of the basic factors concerned in the transmission of malaria. Transactions of the Royal Society of Tropical Medicine and Hygiene. 1955;49(4):339-50.

de Barros FSM, Honorio NA, Arruda ME. Survivorship of Anopheles darlingi (Diptera: Culicidae) in Relation with Malaria Incidence in the Brazilian Amazon. Plos One. 2011;6(8).

dos Santos RL, Forattini OP. Marking-release-recapture methods for determining the size of the natural population of Anopheles albitarsis l.s. (Diptera : Culicidae). Revista De Saude Publica. 1999;33(3):309-13.

Dos Santos RLC, Forattini OP, Burattini MN. Anopheles albitarsis s.l. (Diptera: Culicidae) survivorship and density in a rice irrigation area of the state of São Paulo, Brazil. Journal of Medical Entomology. 2004;41(5):997-1000.

Draper CC, Davidson G. A New Method of Estimating the Survival-Rate of Anopheline Mosquitoes in Nature. Nature. 1953;172(4376):503-.

Epopa PS, Millogo AA, Collins CM, North A, Tripet F, Benedict MQ, et al. The use of sequential mark-release-recapture experiments to estimate population size, survival and dispersal of male mosquitoes of the Anopheles gambiae complex in Bana, a west African humid savannah village. Parasites & Vectors. 2017;10.

Fabian MM, Toma T, Tsuzuki A, Saita S, Miyagi I. Mark-release-recapture experiments with Anopheles saperoi (Diptera: Culicidae) in the Yona Forest, northern Okinawa, Japan. Southeast Asian J Trop Med Public Health. 2005;36(1):54-63.

Fernandez-Salas I, Rodriguez MH, Roberts DR. Gonotrophic cycle and survivorship of Anopheles pseudopunctipennis (Diptera: Culicidae) in the Tapachula foothills of southern Mexico. Journal of Medical Entomology. 1994;31(3):340-7.

Garrett-Jones C, Shidrawi GR. MALARIA VECTORIAL CAPACITY OF A POPULATION OF ANOPHELES GAMBIAE - AN EXERCISE IN EPIDEMIOLOGICAL ENTOMOLOGY. Bull World Health Organ. 1969;40(4):531-+.

Ghavami MB. Estimation and comparison of Anopheles maculipennis s.l. (Diptera: Culicidae) survival rates with light-trap and indoor resting data. Iranian Journal of Public Health. 2005;34(2):48-57.

Gillies MT. Studies on the dispersion and survival of Anopheles gambiae Giles in East Africa, by means of marking and release experiments. Bull Entomol Res. 1961;52(1):99-127.

Gillies MT, Wilkes TJ. OBSERVATIONS ON NULLIPAROUS AND PAROUS RATES IN A POPULATION OF ANOPHELES FUNESTUS IN EAST AFRICA. ANN TROP MED PARASIT. 1963;57:204-13.

Gillies MT, Wilkes TJ. A study of the age-composition of populations of Anopheles gambiae Giles and A. funestus Giles in North-Eastern Tanzania. Bull Entomol Res. 1965;56(2):237-62.

Graves PM, Burkot TR, Saul AJ, Hayes RJ, Carter R. ESTIMATION OF ANOPHELINE SURVIVAL RATE VECTORIAL CAPACITY AND MOSQUITO INFECTION PROBABILITY FROM MALARIA VECTORI INFECTION RATES IN VILLAGES NEAR MADANG, PAPUA NEW GUINEA. J Appl Ecol. 1990;27(1):134-47.

Gunasekaran K. Age composition, natural survival and population growth of Anopheles fluviatilis James, 1902, the major malaria vector in the endemic belt of Koraput District, Orissa, India. The Southeast Asian journal of tropical medicine and public health. 1994;25(1):196-200.

Gunasekaran K, Sahu SS, Jambulingam P. Estimation of vectorial capacity of Anopheles minimus Theobald & An. fluviatilis James (Diptera: Culicidae) in a malaria endemic area of Odisha State, India. Indian Journal of Medical Research. 2014;140:653-9.

Hii JL, Birley MH, Sang VY. Estimation of survival rate and oviposition interval of Anopheles balabacensis mosquitoes from mark-recapture experiments in Sabah, Malaysia. Medical and veterinary entomology. 1990;4(2):135-40.

Hii JLK, Vun YS. A study of dispersal, survival and adult population estimates of the malaria vector, Anopheles balabacensis Baisas (Diptera: Culicidae) in Sabah, Malaysia. Tropical Biomedicine. 1985;2(2):121-31.

Hitchcock JC. AGE COMPOSITION OF A NATURAL POPULATION OF ANOPHELES QUADRIMACULATUS SAY (DIPTERA - CULICIDAE) IN MARYLAND USA. Journal of Medical Entomology. 1968;5(1):125-+.

Hobbs JH, Lowe RE, Schreck CE. Studies of Flight Range and Survival of Anopheles-Albimanus Wiedemann in El-Salvador .1. Dispersal and Survival during Dry Season. Mosquito News. 1974;34(4):389-93.

Jaal Z, Macdonald WW. A MARK RELEASE RECAPTURE EXPERIMENT WITH ANOPHELES-LESTERI-PARALIAE IN NORTHWEST PENINSULAR MALAYSIA. ANN TROP MED PARASIT. 1992;86(4):419-24.

Jensen T, Dritz DA, Fritz GN, Washino RK, Reeves WC. Lake Vera revisited: Parity and survival rates of Anopheles punctipennis at the site of a malaria outbreak in the Sierra Nevada foothills of California. American Journal of Tropical Medicine and Hygiene. 1998;59(4):591-4.

Kenawy MA. Development and Survival of Anopheles-Pharoensis and an-Multicolor from Faiyum, Egypt. Journal of the American Mosquito Control Association. 1991;7(4):551-5.

Khan AQ, Talibi SA. EPIDEMIOLOGICAL ASSESSMENT OF MALARIA TRANSMISSION IN AN ENDEMIC AREA OF EAST PAKISTAN AND SIGNIFICANCE OF CONGENITAL IMMUNITY. Bull World Health Organn. 1972;46(6):783-&.

Krafsur ES. Estimation of the theoretical daily survival rate in some malaria vectors in a lowland region of Ethiopia. Parassitologica. 1970.

Krafsur ES, Garrett-Jones C. The survival in nature of Wuchereia-infected Anopheles funestus Giles in North-eastern Tanzania. Transactions of the Royal Society of Tropical Medicine and Hygiene. 1977;71(2):155-60.

Lee HI, Lee JS, Shin EH, Lee WJ, Kim YY, Lee KR. Malaria transmission potential by Anopheles sinensis in the Republic of Korea. Korean J Parasitol. 2001;39(2):185-92.

Liu QY, Liu XB, Zhou GC, Jiang JY, Guo YH, Ren DS, et al. Dispersal Range of Anopheles sinensis in Yongcheng City, China by Mark-Release-Recapture Methods. Plos One. 2012;7(11).

Loong KP, Chiang GL, Eng KL, Chan ST, Yap HH. Survival and feeding behaviour of Malaysian strain of Anopheles maculatus Theobald (Diptera: Culicidae) and their role in malaria transmission. Tropical Biomedicine. 1990;7(1):71-6.

Lowe R, et al. Studies on flight range and survival of Anopheles albimanus Wiedemann in El Salvador. II. Comparisons of release methods with sterile and normal adults in wet and dry seasons. Mosquito News. 1975;35(2):160-8.

Macdonald G. The analysis of the sporozoite rate. Tropical diseases bulletin. 1952;49(6):569-86.

Mahmood F, Reisen WK. Duration of the Gonotrophic Cycles of Anopheles-Culicifacies Giles and Anopheles-Stephensi Liston, with Observations on Reproductive Activity and Survivorship during Winter in Punjab Province, Pakistan. Mosquito News. 1981;41(1):41-50.

Midega JT, Mbogo CM, Mwambi H, Wilson MD, Ojwang G, Mwangangi JM, et al. Estimating dispersal and survival of Anopheles gambiae and Anopheles funestus along the Kenyan coast by using mark-release-recapture methods. Journal of Medical Entomology. 2007;44(6):923-9.

Milby MM, Reisen WK. Estimation of vectorial capacity: vector survivorship. Bulletin of the Society for Vector Ecology 14(1): 47-54. 1989;14(1):47-54.

Mutero CM, Birley MH. Estimation of the survival rate and oviposition cycle of field populations of malaria vectors in Kenya. J Appl Ecol. 1987;24(3):853-63.

Ndoen E, Wild C, Dale P, Sipe N, Dale M. Mosquito Longevity, Vector Capacity, and Malaria Incidence in West Timor and Central Java, Indonesia. International Scholarly Research Notices. 2012.

Nutsathapana S, Sawasdiwongphorn P, Chitprarop U, Cullen JR, Gass RF, Green CA. A MARK-RELEASE-RECAPTURE DEMONSTRATION OF HOST-PREFERENCE HETEROGENEITY IN ANOPHELES-MINIMUS THEOBALD (DIPTERA, CULICIDAE) IN A THAI VILLAGE. Bull Entomol Res. 1986;76(2):313-20.

Olayemi IK, Ande AT. Survivorship of anopheles gambiae in relation to malaria transmission in Ilorin, Nigeria. Online Journal of Health and Allied Sciences. 2008;7(3).

Pan SQ, Ho KM, Li ST. Preliminary observations on the longevity of males of Anopheles sinensis in natural populations. Annual Bulletin of the Society of Parasitology, Guangdong Province. 1983;4/5:128-30.

Qu C, Yang L, Zhai Y, Shi C, Wang M, Wang Z, et al. The quantitative research of a natural Anopheles sinensis population. Chinese Journal of Parasitic Disease Control. 1997;10(2):137-40.

Quinones ML, Lines JD, Thomson MC, Jawara M, Morris J, Greenwood BM. Anopheles gambiae gonotrophic cycle duration, biting and exiting behaviour unaffected by permethrin-impregnated bednets in The Gambia. Med Vet Entomol. 1997;11(1):71-8.

Quraishi MS, Esghi N, Faghih MA. Flight range, lengths of gonotrophic cycles, and longevity of P-32-labeled Anopheles stephensi mysorensis. Journal of Economic Entomology. 1966;59(1):50-5.

Rawlings P, Curtis CF, Wickramasinghe MB, Lines J. The influence of age and season on dispersal and recapture of Anopheles culicifacies in Sri Lanka. Ecol Entomol. 1981;6(3):307-19.

Ree HI, Hwang UW. Comparative study on longevity of Anopheles sinensis in malarious and non-malarious areas in Korea. Korean Journal of Parasitology. 2000;38(4):263.

Ree HI, Hwang UW, Lee IY, Kim TE. Daily survival and human blood index of Anopheles sinensis, the vector species of malaria in Korea. Journal of the American Mosquito Control Association. 2001;17(1):67-72.

Reisen WK, Aslamkhan M. A release-recapture experiment with the malaria vector, Anopheles stephensi Liston, with observations on dispersal, survivorship, population size, gonotrophic rhythm and mating behaviour. ANN TROP MED PARASIT. 1979;73(3):251-69.

Reisen WK, Mahmood F, Azra K. ANOPHELES-CULICIFACIES GILES - ADULT ECOLOGICAL PARAMETERS MEASURED IN RURAL PUNJAB-PROVINCE, PAKISTAN USING CAPTURE-MARK-RELEASE-RECAPTURE AND DISSECTION METHODS, WITH COMPARATIVE OBSERVATIONS ON ANOPHELES-STEPHENSI LISTON AND ANOPHELES-SUBPICTUS GRASSI. Researches on Population Ecology. 1981;23(1):39-60.

Reisen WK, Mahmood F, Niaz S, Azra K, Parveen T, Mukhtar R, et al. Population-Dynamics of Some Pakistan Mosquitos - Temporal Changes in Reproductive Status, Age Structure and Survivorship of Anopheles-Culicifacies, Anopheles-Stephensi and Culex-Tritaeniorhynchus. ANN TROP MED PARASIT. 1986;80(1):77-95.

Reisen WK, Mahmood F, Parveen T. ANOPHELES-CULICIFACIES GILES - A RELEASE-RECAPTURE EXPERIMENT WITH COHORTS OF KNOWN AGE WITH IMPLICATIONS FOR MALARIA EPIDEMIOLOGY AND GENETIC-CONTROL IN PAKISTAN. Trans R Soc Trop Med Hyg. 1980;74(3):307-17.

Reisen WK, Mahmood F, Parveen T. Anopheles-Subpictus Grassi - Observations on Survivorship and Population-Size Using Mark-Release-Recapture and Dissection Methods. Researches on Population Ecology. 1979;21(1):12-29.

Reisen WK, Mahmood F, Parveen T. Seasonal Trends in Population-Size and Survivorship of Anopheles-Culicifacies, Anopheles-Stephensi and Anopheles-Subpictus (Diptera, Culicidae) in Rural Punjab Province, Pakistan. Journal of Medical Entomology. 1982;19(1):86-97.

Rodriguez MH, Bown DN, Arredondo-Jimenez JI, Villarreal C, Loyola EG, Frederickson CE. Gonotrophic cycle and survivorship of Anopheles albimanus (Diptera: Culicidae) in southern Mexico. Journal of medical entomology. 1992;29(3):395-9.

Shin EH, Lee WJ, Lee HI, Lee DK, Klein TA. Seasonal population density and daily survival of anopheline mosquitoes (Diptera : Culicidae) in a malaria endemic area, Republic of Korea. Journal of Vector Ecology. 2005;30(1):33-40.

Takken W, Charlwood JD, Billingsley PF, Gort G. Dispersal and survival of Anopheles funestus and A-gambiae sl (Diptera : Culicidae) during the rainy season in southeast Tanzania. Bull Entomol Res. 1998;88(5):561-6.

Tanga MC, Ngundu WI, Tchouassi PD. Daily survival and human blood index of major malaria vectors associated with oil palm cultivation in Cameroon and their role in malaria transmission. Tropical Medicine and International Health. 2011;16(4):447-57.

Tavsanoglu N, Caglar SS. The vectoral capacity of Anopleles sacharovi in the malaria endemic area of Sanhurfa, Turkey. European Mosquito Bulletin 2008;26:18-23.

Taye B, Lelisa K, Emana D, Asale A, Yewhalaw D. Seasonal Dynamics, Longevity, and Biting Activity of Anopheline Mosquitoes in Southwestern Ethiopia. Journal of Insect Science. 2016;16.

Toure YT, Dolo G, Petrarca V, Traore SF, Bouare M, Dao A, et al. Mark-release-recapture experiments with Anopheles gambiae sl in Banambani Village, Mali, to determine population size and structure. Med Vet Entomol. 1998;12(1):74-83.

van den A. Daily mortality in four species of New Guinea anophelines. Tropical and geographical medicine. 1959;11:223-36.

Vercruysse J. Estimation of the survival rate of Anopheles arabiensis in an urban area (Pikine--Senegal). J Anim Ecol. 1985;54(2):343.

Weathersbee AA, Meisch MV. Parous and Survival Rate Estimates for Anopheles-Quadrimaculatus (Diptera, Culicidae) in the Arkansas Grand Prairie. Environ Entomol. 1991;20(6):1595-600.

Zaim M, Zahirnia AH, Manouchehri AV. Survival Rates of Anopheles-Culicifacies S L and Anopheles-Pulcherrimus in Sprayed and Unsprayed Villages in Ghassreghand District, Baluchistan, Iran, 1991. Journal of the American Mosquito Control Association. 1993;9(4):421-5.
