# Supplementary figures and images for: An overview of malarial Anopheles mosquito survival estimates in relation to methodology
Source: Parasit Vectors. 2020 May 7;13:233. doi: 10.1186/s13071-020-04092-4 (PMC7206813; doi:10.1186/s13071-020-04092-4)

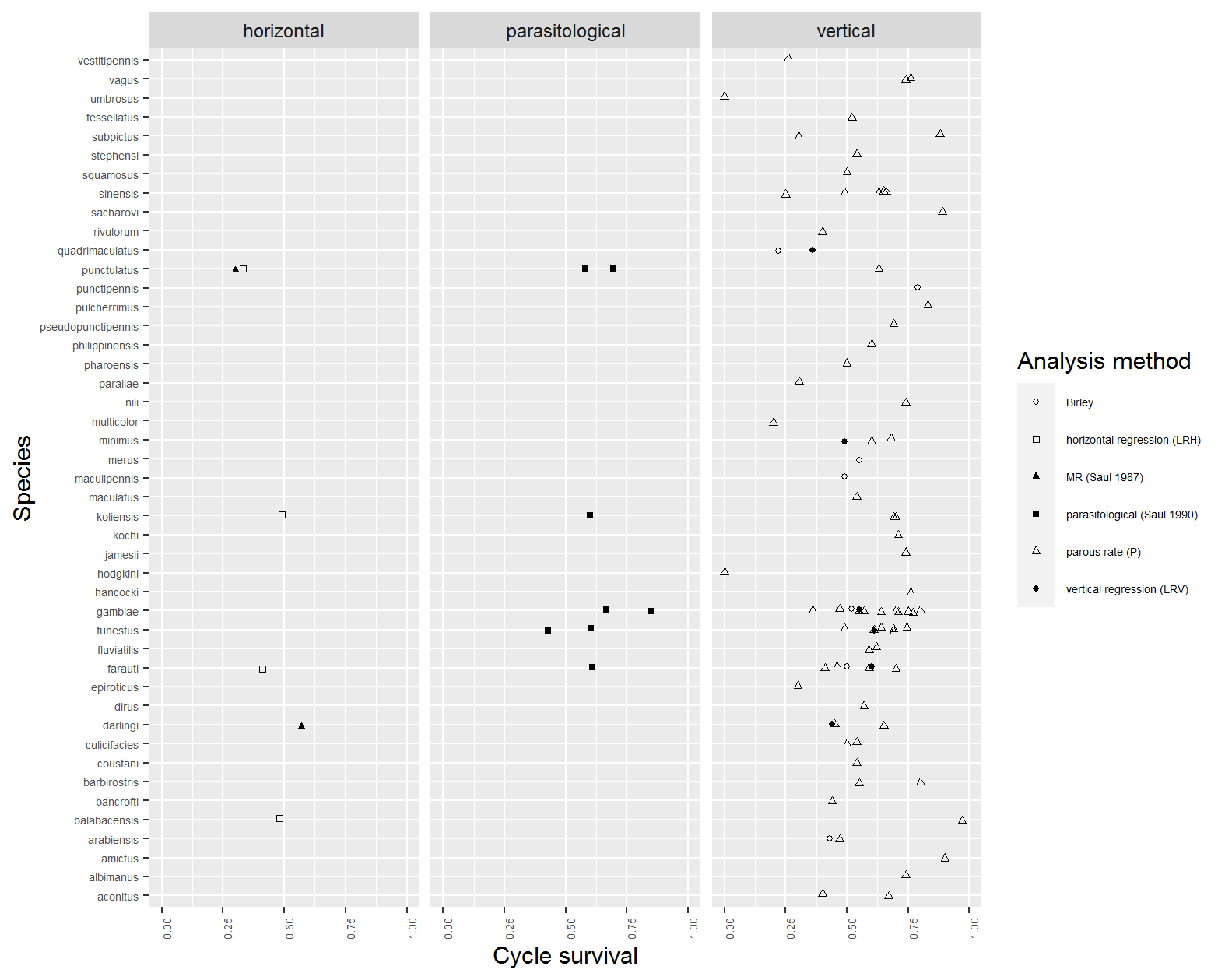

Supplement: Supplementary file 2 — Additional file 2: Figure S1: Probability of cycle survival by Anopheles species and by method (vertical, horizontal or parasitological) and analysis. [file 13071_2020_4092_MOESM2_ESM.tiff]
